# A neural network-based automatic semi-variogram modeling approach for geomagnetic map construction in multi-source indoor and outdoor navigation

**DOI:** 10.1038/s41598-025-31721-8

**Published:** 2025-12-10

**Authors:** Chengsheng Zhan, Ping Huang, Bing Xue, Bin Lan

**Affiliations:** 1 College of Intelligent Systems Science and Engineering, Harbin Engineering University, Harbin, 150001 China; 2 Jianghuai Advance Technology Center, Hefei, 230000 China

**Keywords:** Engineering, Mathematics and computing, Solid Earth sciences

## Abstract

High-precision geomagnetic maps are essential for geomagnetic-assisted navigation, yet their construction is constrained by kriging interpolation’s reliance on accurately modeled semi-variogram. Conventional approaches depend heavily on geological expertise, introducing subjectivity and limiting both mapping accuracy and navigation performance. Here, we present geomagnetic map via auto-semi-variogram kriging(GMAS-K), a framework that integrates geomagnetic map via auto-semi-variogram convolutional neural network(GMAS-CNN) to automatically infer semi-variogram parameters. GMAS-CNN adopts an encoder–decoder architecture: the encoder compresses and fuses multi-scale features of geomagnetic samples to enrich semi-variance representations, while the decoder reconstructs latent feature spaces to estimate semi-variogram parameters. To further enhance cross-scale consistency, we introduce a multiple convolutional block attention module (M-CBAM). Experiments show that GMAS-K surpasses ordinary kriging, producing smoother and more accurate geomagnetic maps while streamlining the mapping workflow. These results highlight the promise of coupling deep learning with geostatistical interpolation to advance geomagnetic mapping and improve navigation accuracy.

## Introduction

**Fig. 1:**
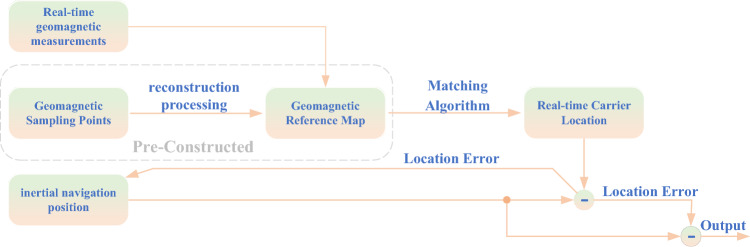
Geomagnetic navigation process.

Geomagnetic-assisted navigation has attracted increasing interest in both indoor and outdoor environments due to its passive operation, all-weather availability, robustness to environmental factors, immunity to non-line-of-sight constraints, and strong resistance to interference^1–6^. It is typically deployed as part of multi-source fusion navigation, with the overall workflow illustrated in Fig. 1. The process generally comprises three steps: constructing a high-precision geomagnetic reference map, applying geomagnetic matching algorithms, and correcting inertial navigation errors^7^. Among these, the reference map is pivotal, as it directly determines navigation accuracy. Two main strategies are used to construct geomagnetic maps: model-based approaches^8,9^ and interpolation-based methods^10^. Model-based approaches often yield low-resolution maps that fail to meet the requirements of high-accuracy navigation. As a result, geomagnetic reference maps are usually generated by combining sparse in-situ measurements with interpolation techniques, with kriging being the most widely adopted.

The use of kriging interpolation in geomagnetic map construction has been extensively studied. Du et al. applied ordinary kriging to magnetic measurements, detailing the interpolation process^11^. Wang et al. proposed an exponentially weighted particle filter to estimate object pose distributions, incorporating kriging to update magnetic field maps^12^. Li et al. analyzed the influence of anisotropy in sparse geomagnetic samples on interpolation error and introduced a correction method that rotates and stretches sampling points to mitigate anisotropy and improve kriging accuracy^13^. Ji et al. developed the bidirectional variability interpolation method (BVPAO-K), which applies kriging independently along longitude and latitude before merging the results^14^. Although these methods improve interpolation accuracy and map resolution, they face a critical limitation: the difficulty of accurately modeling the semi-variogram, which in turn increases errors when applying kriging for geomagnetic map construction^15^. Constructing an appropriate semi-variogram requires substantial expertise in geology and geostatistics, which many researchers lack. Consequently, errors in semi-variogram modeling propagate through the interpolation process, degrading kriging performance. This challenge underscores the need for automated algorithms capable of directly estimating semi-variogram parameters from sparse geomagnetic data.

To bridge this gap, researchers have begun exploring neural networks for geomagnetic mapping. Ma et al. proposed a convolutional neural network (CNN) for constructing geomagnetic reference maps^16^, although their method requires three-component geomagnetic inputs that are often impractical to obtain. Neural networks have also been employed to estimate semi-variogram parameters by exploiting their strong feature-extraction capabilities. Soulaimani et al. coupled Bayesian optimization with neural networks for semi-variogram modeling, substantially improving parameter-estimation accuracy^17^. Erten et al. assessed two direct machine-learning approaches that bypass semi-variograms; used in isolation, neither outperformed ordinary kriging, underscoring the central role of semi-variogram modeling^18^. Wang et al. proposed a spatiotemporal water-quality prediction framework that fuses time-series forecasting with spatial interpolation; a recurrent neural network inferred semi-variogram parameters and markedly improved predictive accuracy^19^. Yang et al. used deep neural networks (DNNs) to infer semi-variogram parameters for kriging-based elevation mapping^20,21^, but their approach was restricted to isotropic conditions and did not address anisotropy. Jo et al. applied neural networks to derive semi-variogram parameters from sparse wellbore pore data for geological prediction^22–24^. Similarly, Mokdad et al. leveraged synthetic sequential Gaussian simulation realizations (SGSR) as neural network inputs for semi-variogram estimation at specified lag distances^25^. Collectively, these studies demonstrate the promise of neural networks in automating semi-variogram modeling, but their application to anisotropic geomagnetic mapping remains largely unexplored.

Inspired by the work of Honggeun Jo, Karim Mokdad, and others in the oil well domain on automatic semi-variogram inference, and motivated by the need for accurate, de-empirical estimation of semi-variogram parameters in geomagnetic mapping, we propose a novel framework that integrates automatic semi-variogram inference with kriging interpolation to generate high-precision geomagnetic maps. Specifically, we introduce geomagnetic map via automated semi-Variogram inference network(GMAS-CNN), an encoder–decoder architecture. The encoder extracts multi-scale convolutional features to enrich covariance representations, while the decoder reconstructs latent features to infer semi-variogram parameters. To enhance cross-scale consistency, we incorporate a multiple convolutional block attention module (M-CBAM), improving both spatial and channel-wise feature robustness.Our contributions are threefold:

1. We propose GMAS-K, a framework that combines neural network-based semi-variogram inference with kriging interpolation to enable automated, high-precision geomagnetic mapping.
2. We introduce GMAS-CNN, a novel encoder-decoder architecture augmented with M-CBAM, specifically designed to accurately infer semi-variogram parameters from sparse and irregularly distributed geomagnetic samples.
3. We validate our approach on datasets from multiple real-world sampling sites, demonstrating that GMAS-K outperforms ordinary kriging interpolation methods in both accuracy and robustness, while providing a streamlined, automated workflow.

## Materials and methods

Kriging interpolation, originally proposed by South African mining engineer Danie G. Krige^26^, was developed to estimate mineral deposit distributions in mining exploration. Mathematically, kriging is linear, unbiased, and optimal, making it a powerful tool for enhancing the resolution of geomagnetic maps derived from sparse measurements.

The semi-variogram quantifies spatial autocorrelation in the data and assesses the similarity between neighboring points, forming the basis of kriging interpolation. Following the first law of geography—“nearby points are more similar than distant ones”—the semi-variogram is defined as:

1 $$\begin{aligned} \begin{aligned} \gamma (l)&= \dfrac{1}{2}\,\textrm{Var}\!\left[ Z(x) - Z(x+l) \right] \\&= \dfrac{1}{2}\left\{ \mathbb {E}\!\left[ \left( Z(x)-Z(x+l)\right) ^{2} \right] - \left( \mathbb {E}\!\left[ Z(x)-Z(x+l) \right] \right) ^{2} \right\} \\&= \dfrac{1}{2}\,\mathbb {E}\!\left[ \left( Z(x)-Z(x+l)\right) ^{2} \right] , \end{aligned} \end{aligned}$$

where $\gamma (l)$ denotes the semi-variogram value between points separated by distance *l*, and *Z*(*x*) and $Z(x+l)$ are the geomagnetic values at locations *x* and $x+l$, respectively. In this paper, the spherical model is adopted as the semi-variogram function.

In this paper, We present a neural network-based method that automatically infers semivariogram parameters and integrates them with kriging to generate high-precision geomagnetic maps. The framework comprises three key steps: first, multiple training datasets are generated from sparse geomagnetic samples with known semi-variogram parameters; next, a neural network is designed to infer these parameters; finally, kriging interpolation is applied to construct the geomagnetic map.

### Generating geomagnetic semi-variogram automatic inference network training data

**Fig. 2:**
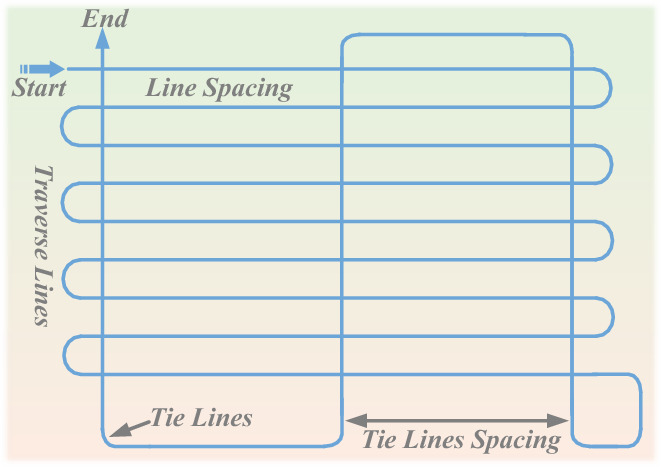
Geomagnetic sampling route.

To generate the training dataset, geomagnetic measurement samples are required. Regional sampling is typically conducted using airborne magnetic measurement systems, producing sparse geomagnetic values after data processing. Typical survey routes for geomagnetic carriers^1^ are illustrated in Fig. 2, consisting of measurement lines and connecting lines. In this paper, points from both types of lines are used, in combination with sequential gaussian simulation (SGS), to generate the training dataset. For SGS-based dataset generation, the range of semi-variogram parameters must first be determined, and sampling points are selected from both measurement and connecting lines accordingly.

The sparse geomagnetic values used in this study are drawn from MagNav, an open-access dataset released as part of a global challenge to advance AI-enabled geomagnetic navigation^27^. The dataset was collected primarily by Sanders Geophysics Ltd. (SGL) using a Cessna 208B Grand Caravan—a standard platform for geological surveys—and comprises 34h46min of flight data acquired in 2020–2021, including three-component and scalar geomagnetic measurements, aircraft attitude and altitude, GPS, and electrical currents from multiple onboard instruments. We constructed from these data a geomagnetic map providing positions with corresponding magnetic-intensity values. To assess the generalization of our network, we additionally used the Residual Total Magnetic Field, Kluane Lake West Aeromagnetic Survey, Yukon, NTS115-G/2 and 3 dataset, collected by two AS350 helicopters equipped with HM1 systems and instrumentation including radar, laser, and barometric altimeters, cameras, digital recorders, electronic navigation equipment, dual-frequency GPS receivers, magnetometer sensors, and optically pumped magnetometers. From the resulting maps, we extracted two geographically distinct sets of sample points to evaluate performance; results are presented in the section “Test of results of geomagnetic map construction.”

**Fig. 3:**
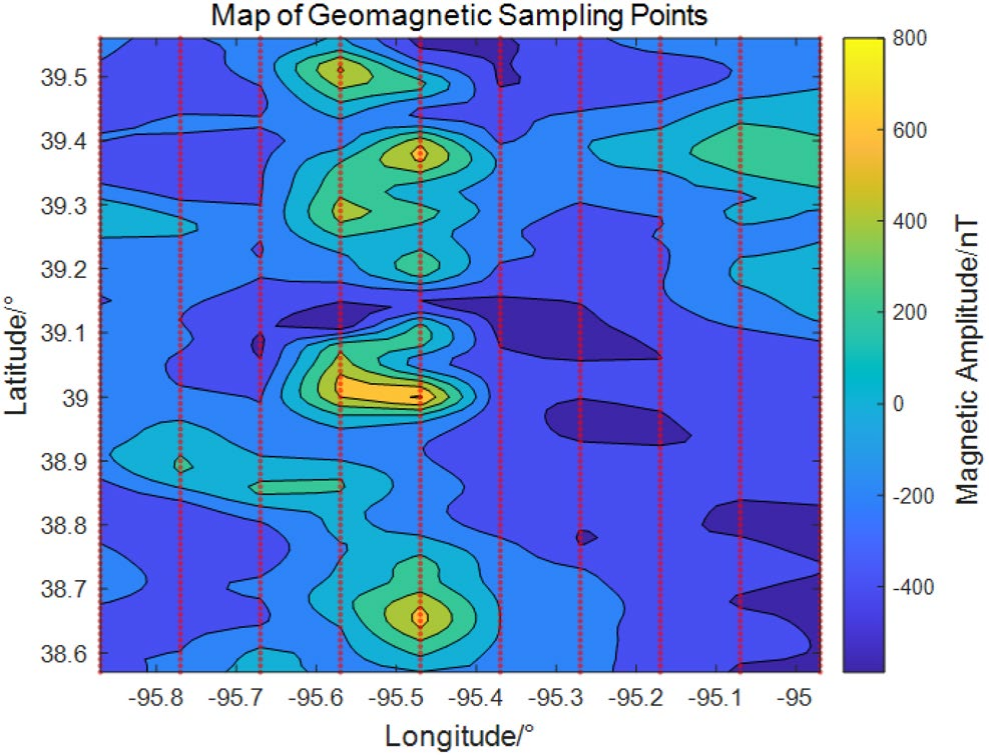
Geomagnetic sample point sampling locations and corresponding geomagnetic values.

The sampling locations are shown in Fig. 3. To prepare the data for the proposed network, geomagnetic values are first transformed using a normal score transformation^28^ and subsequently normalized. After kriging interpolation, an inverse transformation is applied to restore the physical units of the data.

**Fig. 4:**
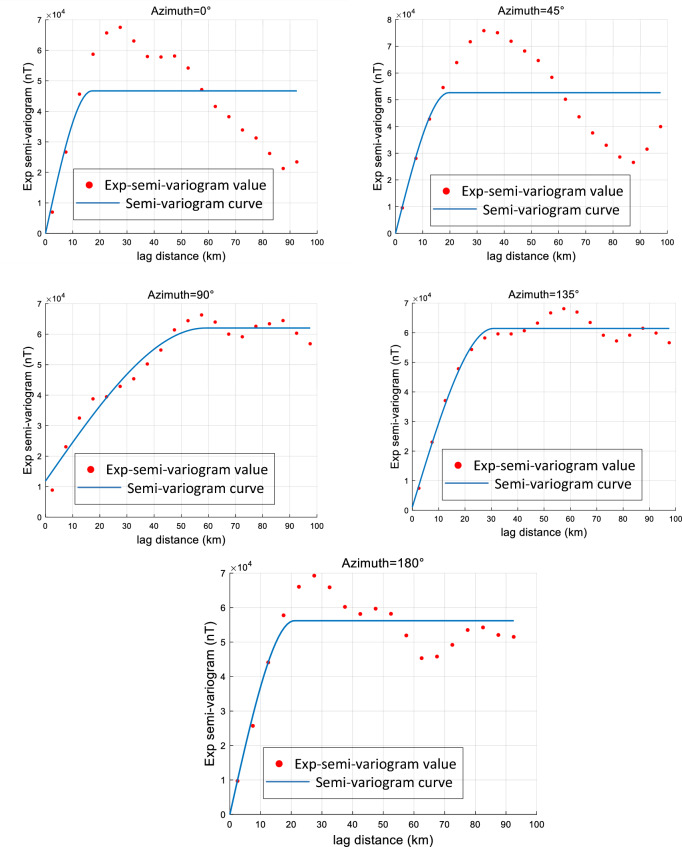
Semi-variogram curves for each experience in the 0, 45, 90, 135 and 180 directions.

After obtaining the sample points, the approximate range of the semi-variogram must be determined to generate the training dataset. This is typically achieved by computing empirical semi-variogram to estimate the parameter ranges for the geomagnetic data. The geomagnetic map is analyzed along five directions ($0^\circ$, $45^\circ$, $90^\circ$, $135^\circ$, and $180^\circ$), and for each direction, the empirical semi-variogram is calculated. These empirical values are then fitted using the least squares method, and scatter plots of the distances are generated. The resulting semi-variogram curves and parameters are shown in Fig. 4, where the scatter points represent empirical values at varying lag distances. Based on the scatter distributions and least squares fitting, the semi-variogram ranges used for dataset generation are summarized in Table 1.

The semi-variogram parameter ranges, summarized in Table 1, are as follows: the azimuth of the major direction spans $0^\circ -180^\circ$; the range along the major direction is 15-85 km; anisotropy ratios vary from 1 to 4.8; nugget values range from 0 to 0.4; and sills range from 0.6 to 1. The geomagnetic field is primarily modeled using spherical semi-variograms, yielding a total of 14,400 possible parameter combinations.

**Table 1:** Detailed information on the parameters of the semi-variogram model used to train the CNN model.

semi-variogram parameters	Range of values	Step
Azimuth of major direction	0–180($^\circ$)	10($^\circ$)
Range in the major direction	15–85 (km)	10 (km)
Anisotropy ratio	1–4.8	0.2
Nugget effect	0–0.4	0.1

After determining the semi-variogram parameter ranges from the actual sample points, the training dataset is generated using the SGS method. Let the set of sampled geomagnetic points be:

2 $$\begin{aligned} \left\{ \text {mag}_0(x_0, y_0), \text {mag}_1(x_1, y_1), \text {mag}_2(x_2, y_2), \dots , \text {mag}_n(x_n, y_n) \right\} , \end{aligned}$$

where $(x_i,y_i)$ represents the *i*-th position of the sample point, and $mag_i$ represents the corresponding geomagnetic value. The semi-variogram parameters for the training dataset are:

3 $$\begin{aligned} \left\{ f_0(\theta _0, a_0, l_0, c_0^0), f_1(\theta _1, a_1, l_1, c_1^0), f_2(\theta _2, a_2, l_2, c_2^0), \dots , f_n(\theta _n, a_n, c_n^0) \right\} , \end{aligned}$$

where $\theta _i$ is the direction angle of the *i*-th semi-variogram, $a_i$ is the maximum range, $l_i$ is the anisotropy ratio, $c_i^0$ denotes the nugget value, and $f_i$ represents the *i*-th semi-variogram function.

Using the SGS method, the corresponding SGS realizations (SGSR) are generated as:

4 $$\begin{aligned} SGSR_i = F\left( f_i(\theta _i, a_i, l_i, c_i^0); \left( \text {mag}_0(x_0, y_0), \text {mag}_1(x_1, y_1), \dots , \text {mag}_n(x_n, y_n) \right) \right) , \end{aligned}$$

where *F* denotes the SGS mapping function. Each combination of semi-variogram parameters with the SGS mapping produces a specific SGSR, capturing the full semi-variogram distribution of the real geomagnetic field and forming a comprehensive training dataset. For each SGSR, sampling is performed at the same locations as the original measurements. Each realization is sampled at $100 \times 10$ points as inputs to the encoder, with the corresponding SGSR surface serving as the label. The encoder outputs SGSR estimates matching the dimensionality of the original surfaces, and the decoder subsequently processes these outputs to infer semi-variogram parameters from sparse points. These inferred parameters are then used in kriging interpolation to construct high-precision geomagnetic maps.

**Fig. 5:**
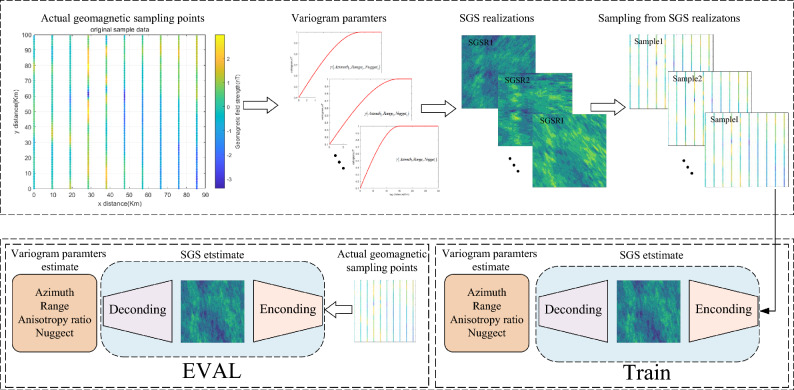
GMAS-CNN training flowchart.

During training, the mean squared error (MSE) is iteratively minimized, with network weights updated using the ADAM optimizer^29^ until the outputs converge to the labels. Once trained, the network can directly predict the anisotropy angle, maximum range, anisotropy ratio, and nugget value from sparse geomagnetic samples. The overall workflow of the method is illustrated in Fig. 5.

### GMAS-CNN network design

**Fig. 6:**
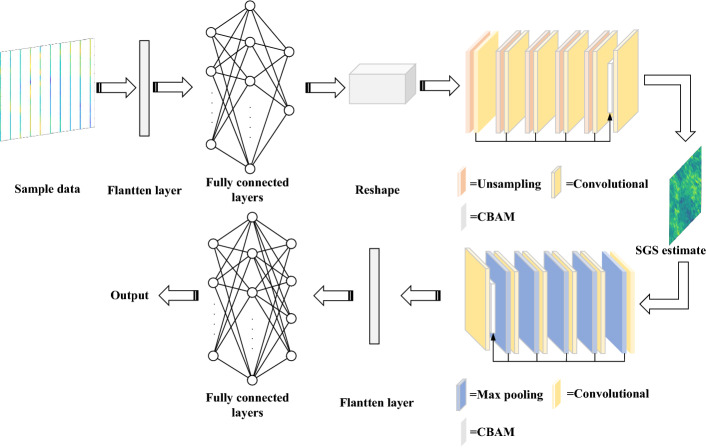
GMAS-CNN network structure diagram.

To predict semi-variogram parameters from geomagnetic sample points, we propose GMAS-CNN, a neural network based on a encoder-decoder architecture, as illustrated in Fig. 6. The network comprises a fully connected layer, 10 convolutional units, 5 upsampling layers, and 5 pooling layers. Each convolutional unit consists of a convolutional layer followed by batch normalization (BN), using a $3 \times 3$ kernel for dimensionality reduction.

In the encoder, upsampling operations are combined with $3 \times 3$ convolutional units to reconstruct geomagnetic sample points into two-dimensional feature maps matching the decoder’s dimensionality. The ecoder integrates multi-scale features via convolutional and pooling layers, facilitating effective extraction and fusion of high-level representations. High-dimensional features capture global planar information and overall structural trends, while low-dimensional features preserve fine-grained local details, reflecting rapid spatial variations.

**Fig. 7:**
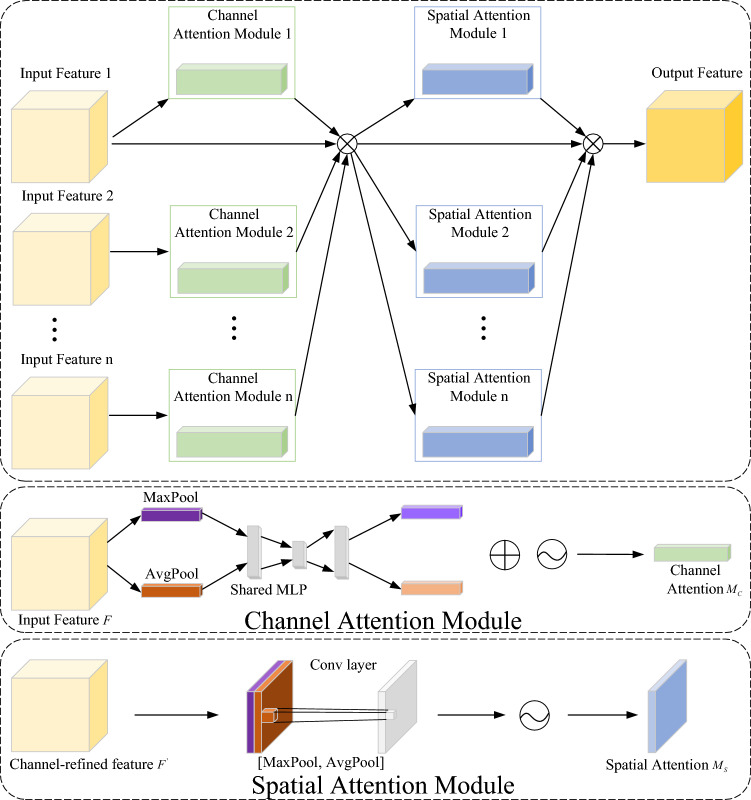
The M-CBAM attention mechanism module proposed in this paper.

To further enhance feature representation, we introduce the M-CBAM attention module, which processes multi-scale convolutional outputs, as illustrated in Fig. 7. The channel-attention module processes multiscale semivariogram features from geomagnetic samples, mitigating feature loss and distortion during changes in dimensionality. A spatial attention module subsequently fuses these features, ensuring strong cross-scale consistency. The M-CBAM^30^ formulation is as follows:

5 $$\begin{aligned} \begin{aligned} M_{C}(F_{1}, F_{2}, \ldots , F_{n})&= \sigma \!\left( W_{1}\!\left( W_{2}\!\left( \operatorname {AvgPool}(F_{1}, F_{2}, \ldots , F_{n})\right) \right) \right) \\&\quad + W_{1}\!\left( W_{2}\!\left( \operatorname {MaxPool}(F_{1}, F_{2}, \ldots , F_{n})\right) \right) \\&= \sigma \!\left( W_{1}\!\left( W_{2}\!\left( F^{C}_{\textrm{Avg}1}, F^{C}_{\textrm{Avg}2}, \ldots , F^{C}_{\textrm{Avgn}}\right) \right) \right) \\&\quad + W_{1}\!\left( W_{2}\!\left( F^{C}_{\textrm{Max}1}, F^{C}_{\textrm{Max}2}, \ldots , F^{C}_{\textrm{Maxn}}\right) \right) , \end{aligned} \end{aligned}$$

6 $$\begin{aligned} \begin{aligned} M_{S}(F_{1}, F_{2}, \ldots , F_{n})&= \sigma \, f \left( \begin{bmatrix} \text {AvgPool}(F_{1}, F_{2}, \ldots , F_{n}) \\ \text {MaxPool}(F_{1}, F_{2}, \ldots , F_{n}) \end{bmatrix} \right) \\&= \sigma \, f \left( \begin{bmatrix} F^{S}_{\text {Avg1}} + F^{S}_{\text {Avg2}} + \cdots \\ + F^{S}_{\text {Avgn}} \\ F^{S}_{\text {Max1}} + F^{S}_{\text {Max2}} + \cdots \\ + F^{S}_{\text {Maxn}} \end{bmatrix} \right) , \end{aligned} \end{aligned}$$

7 $$\begin{aligned} F' = M_{C} \cdot M_{S} \cdot (F_{1} + F_{2} + \cdots + F_{n}), \end{aligned}$$

where $M_C$ and $M_S$ denote the channel and spatial attention parameters, respectively; $F_i$ represents multi-scale input features, and $F'$ is the fused output.

The detailed architecture of GMAS-CNN, including parameter settings, is summarized in Tables 2 and 3. In this design, the encoder employs the leaky_ReLU activation function, while the decoder uses ReLU. Leaky_ReLU preserves negative values during training, enhancing the network’s representational capacity. Furthermore, batch normalization^31^ is applied after each convolutional layer, standardizing features to zero mean and unit variance, which stabilizes and accelerates training.

**Table 2:** GMAS-CNN encoder structure parameters.

Layer type	Dimension	Kernel	Stride	Activate	Trainable
	(width $\times$ height $\times$ channel)	size		Function	Parameters
Input data	1$\times$100$\times$10	–	–	–	–
Flatten	1000	–	–	–	–
Fully connected	1024	–	–	Leaky_ReLU	$W_{1}^{1}, B_{1}^{1}$
Reshape	8$\times$8$\times$16	–	–	–	–
Upsampling	16$\times$16$\times$16	–	–	–	–
Convolution	16$\times$16$\times$16	3$\times$3	1	Leaky_ReLU	$W_{2}^{1}, B_{2}^{1}$
Batch normalization	16$\times$16$\times$16	–	–	–	–
Upsampling	32$\times$32$\times$16	–	–	–	–
Convolution	32$\times$32$\times$16	3$\times$3	1	Leaky_ReLU	$W_{3}^{1}, B_{3}^{1}$
Batch normalization	32$\times$32$\times$16	–	–	–	–
Upsampling	64$\times$64$\times$16	–	–	–	–
Convolution	64$\times$64$\times$16	3$\times$3	1	Leaky_ReLU	$W_{4}^{1}, B_{4}^{1}$
Batch normalization	64$\times$64$\times$16	–	–	–	–
Upsampling	64$\times$64$\times$8	–	–	–	–
Convolution	64$\times$64$\times$8	3$\times$3	1	Leaky_ReLU	$W_{5}^{1}, B_{5}^{1}$
Batch normalization	64$\times$64$\times$8	–	–	–	–
Upsampling	128$\times$128$\times$8	–	–	–	–
Convolution	128$\times$128$\times$4	3$\times$3	1	Leaky_ReLU	$W_{6}^{1}, B_{6}^{1}$
Batch normalization	128$\times$128$\times$4	–	–	–	–
M-CBAM	–	–	–	Leaky_ReLU	$Wca^{1}, Bsa^{1}$
Convolution	128$\times$128$\times$1	3$\times$3	1	Leaky_ReLU	$W_{7}^{1}, B_{7}^{1}$
SGS estimate	128$\times$128$\times$1	–	–	–	–

Within the GMAS-CNN framework, each component contains learnable parameters, including convolutional kernel weights, biases, and fully connected layer weights and node biases. The weight parameters for the encoder and decoder are defined as:

8 $$\begin{aligned} {\begin{matrix} P^{1} = \{ & W_{1}^{1}, W_{2}^{1}, W_{3}^{1}, \dots , W_{7}^{1}, \\ & B_{1}^{1}, B_{2}^{1}, B_{3}^{1}, \dots , B_{7}^{1}; W_{ca}^{1}, W_{sa}^{1} \}, \end{matrix}} \end{aligned}$$

9 $$\begin{aligned} {\begin{matrix} P^{2} = \{ & W_{1}^{2}, W_{2}^{2}, W_{3}^{2}, \dots , W_{7}^{2}, \\ & B_{1}^{2}, B_{2}^{2}, B_{3}^{2}, \dots , B_{7}^{2}; W_{ca}^{2}, W_{sa}^{2} \}, \end{matrix}} \end{aligned}$$

where $P^1$ and $P^2$ denote the encoder and decoder network parameters, respectively. $W_i^1$ and $W_i^2$ represent the weights of convolutional or fully connected layers, while $B_i^1$ and $B_i^2$ are the corresponding biases. $W_{ca}^1$, $W_{sa}^1$, $W_{ca}^2$, and $W_{sa}^2$ correspond to the channel- and spatial-attention parameters of the M-CBAM modules in the encoder and decoder, respectively.

**Table 3:** GMAS-CNN decoder structure parameters.

Layer type	Dimension	Kernel	Stride	Activate	Trainable
	(width $\times$ height $\times$ channel)	size		function	parameters
SGS estimate	128$\times$128$\times$1	–	–	–	–
Convolution	128$\times$128$\times$8	3$\times$3	1	ReLU	$W_{1}^{2},B_{1}^{2}$
Max pooling	64$\times$64$\times$8	–	–	–	–
Convolution	64$\times$64$\times$16	3$\times$3	1	ReLU	$W_{2}^{2},B_{2}^{2}$
Max pooling	32$\times$32$\times$16	–	–	–	–
Convolution	32$\times$32$\times$32	3$\times$3	1	ReLU	$W_{3}^{2},B_{3}^{2}$
Max pooling	16$\times$16$\times$32	–	–	–	–
Convolution	16$\times$16$\times$64	3$\times$3	1	ReLU	$W_{4}^{2},B_{4}^{2}$
Max pooling	8$\times$8$\times$64	–	–	–	–
Convolution	8$\times$8$\times$128	3$\times$3	1	ReLU	$W_{5}^{2},B_{5}^{2}$
Max pooling	4$\times$4$\times$128	–	–	–	–
M-CBAM	4$\times$4$\times$128	1	–	ReLU	$Wca^{2},Bsa^{2}$
Convolution	4$\times$4$\times$128	1	–	ReLU	$W_{6}^{2},B_{6}^{2}$
Dropout	4$\times$4$\times$128	–	–	–	–
Flatten	2048	–	–	–	–
Fully connected	4	–	–	–	$W_{7}^{2},B_{7}^{2}$
Output	4	–	–	–	–

During training, optimal network parameters are obtained by minimizing a loss function. In this paper, the MSE is used:

10 $$\begin{aligned} L(P) = \frac{1}{N} \sum _{i=1}^{N} \left( y_i - \hat{y}_i \right) ^{2}, \end{aligned}$$

where *N* is the number of training samples per batch, $y_i$ is the true semi-variogram parameter, and $\hat{y}_i$ is the network estimate. The loss is backpropagated to iteratively update the model until convergence, yielding optimal network coefficients. To account for inherent noise in geomagnetic measurements, the Adam optimizer is employed for stable convergence under gradient perturbations. A dynamic learning rate is applied, starting at 0.0001 and gradually decreasing over epochs to suppress loss oscillations in later training stages.

### Kriging interpolation

**Fig. 8:**
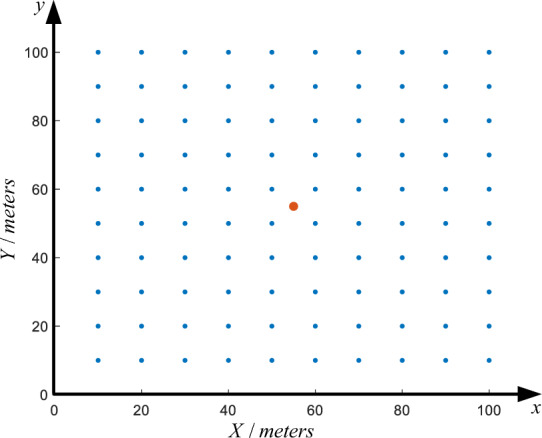
Kriging interpolation diagram.

Once GMAS-CNN has inferred the semi-variogram parameters from geomagnetic sample points, a high-resolution geomagnetic map is generated using kriging interpolation. The process is illustrated in Fig. 8, where blue dots indicate the actual sampling points and red dots denote the interpolation locations.

Through aeromagnetic surveys, samples $Z_1(x_1, y_1), Z_2(x_2, y_2), \dots , Z_n(x_n, y_n)$ are collected within a region, where $Z_i$ denotes the measured geomagnetic value and $(x_i, y_i)$ the sampling coordinates. The value at an interpolation point $(x_o, y_o)$ is denoted as $\hat{Z}_o$, the estimated geomagnetic value. Centered at $(x_o, y_o)$, a search is performed within a specified radius to identify neighboring samples, which are assigned weights $\lambda _1, \lambda _2, \dots , \lambda _n$. The estimated value at the interpolation point is then computed as:

11 $$\begin{aligned} \hat{Z}_{o} = \sum _{i=1}^{n} \lambda _{i} Z_{i}. \end{aligned}$$

The interpolation weights $\lambda _i$ are obtained by solving the kriging system:

12 $$\begin{aligned} \begin{bmatrix} r_{11} & r_{12} & \cdots & r_{1n} & 1 \\ r_{21} & r_{22} & \cdots & r_{2n} & 1 \\ \vdots & \vdots & \ddots & \vdots & \vdots \\ r_{n1} & r_{n2} & \cdots & r_{nn} & 1 \\ 1 & 1 & \cdots & 1 & 0 \end{bmatrix} \begin{bmatrix} \lambda _1 \\ \lambda _2 \\ \vdots \\ \lambda _n \\ -\phi \end{bmatrix} = \begin{bmatrix} r_{1o} \\ r_{2o} \\ \vdots \\ r_{no} \\ 1 \end{bmatrix}, \end{aligned}$$

where $r_{ij}$ denotes the semi-variogram values between sample point pairs, $\phi$ is the Lagrange multiplier, and $r_{io}$ ($i=1, \dots , n$) represents the semi-variogram values between sample points and the interpolation point. Once the semi-variogram model is established and $r_{io}$ computed, the weights $\lambda _i$ can be determined, completing the interpolation process.

## Results

The dataset is fed into the designed GMAS-CNN network for training. By monitoring the network’s loss, the optimal training parameters are determined and saved for inference. Finally, kriging interpolation is applied to construct the high-precision geomagnetic reference map.

### Network testing of geomagnetic semi-variogram automated inference models

The 14,400 generated SGSR surfaces are split into training and test sets at a 9:1 ratio to facilitate training and evaluation of the proposed automatic geomagnetic semi-variogram inference model. Once trained, the network infers semi-variogram parameters directly from in situ geomagnetic measurements, which are subsequently used with kriging to construct high-resolution geomagnetic maps.

#### GMAS-CNN performance testing

**Fig. 9:**
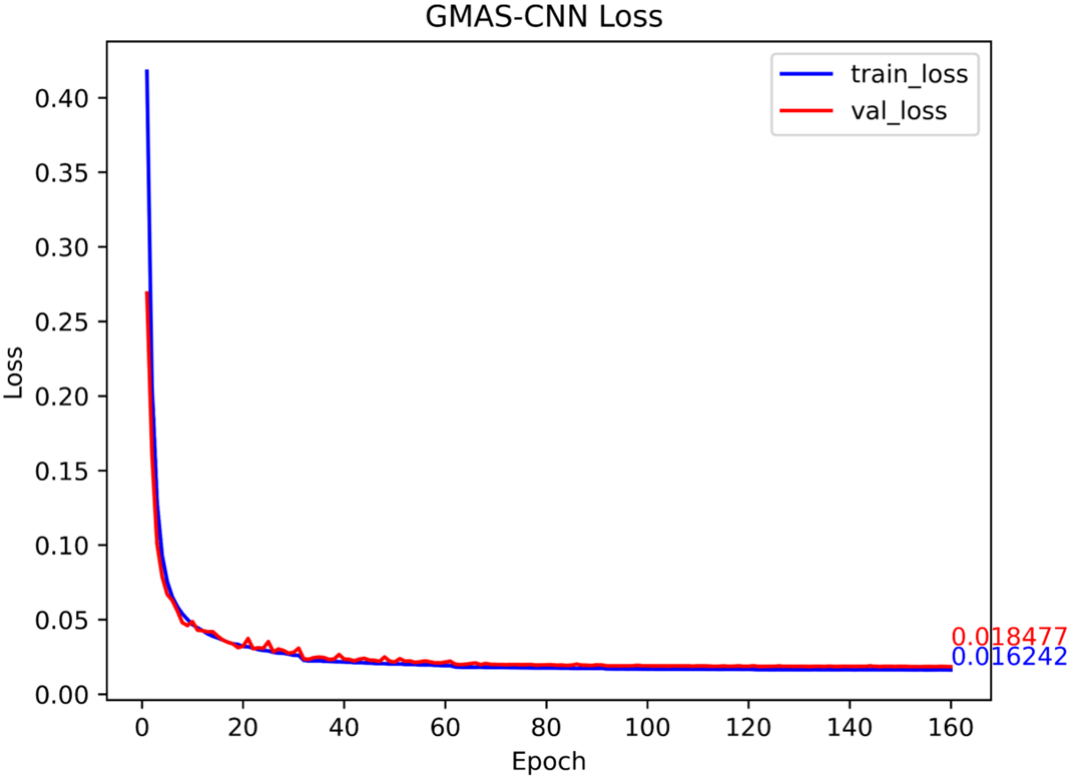
GMAS-CNN network training set and test set loss changes with epoch.

The dataset is fed into the network for training, and the neural network’s performance is evaluated by monitoring the loss function. Figure 9 shows the training and testing loss curves over 160 epochs. The GMAS-CNN loss decreases rapidly before stabilizing, with training and testing curves following consistent trends, indicating the absence of overfitting. During training, the minimum loss value is recorded, and the corresponding network parameters are saved.

**Fig. 10:**
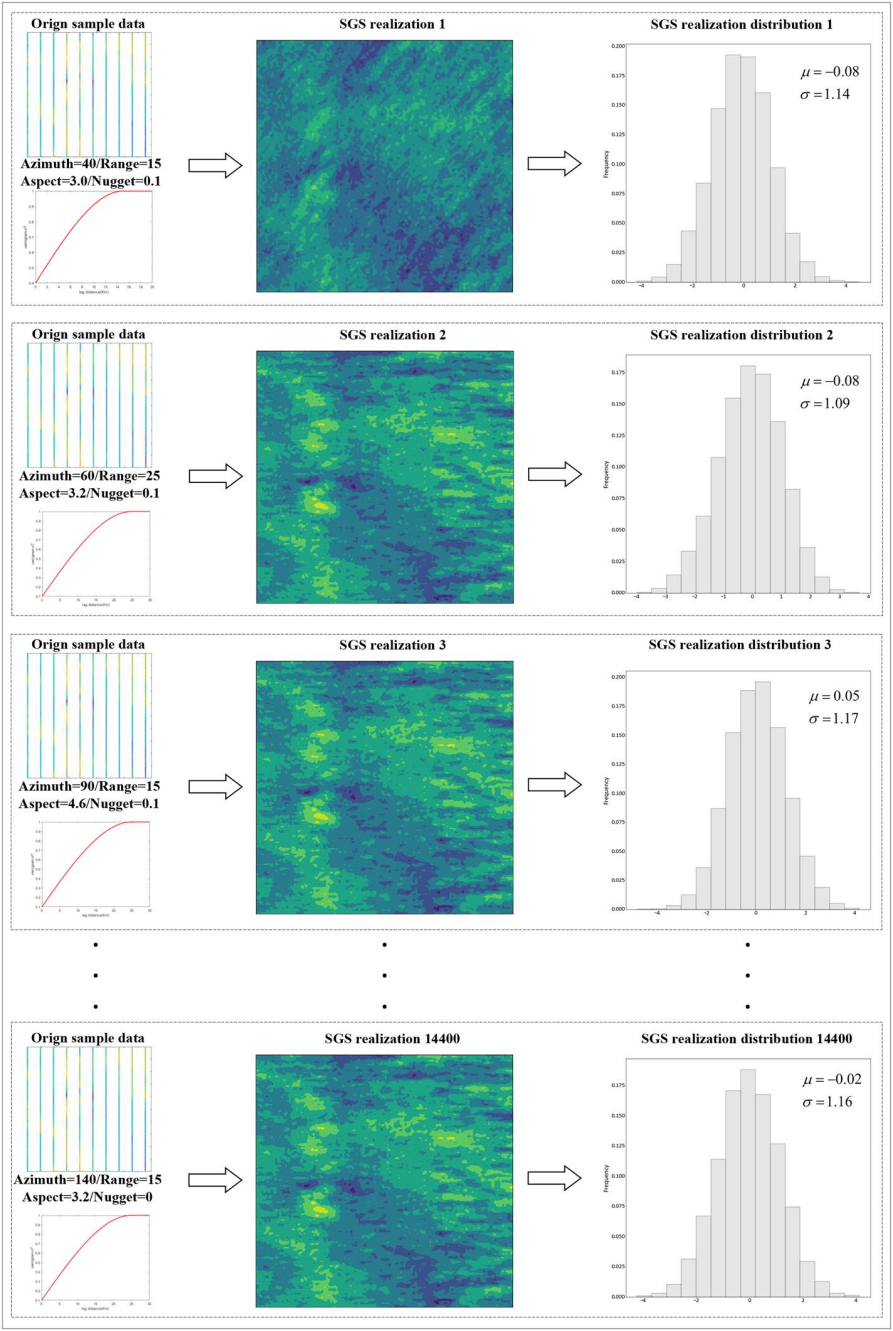
Sample points generate SGSR and corresponding histogram distributions through the SGS process.

First, the generated SGS dataset is analyzed, as shown in Fig. 10, comprising a total of 14,400 training samples. The first column displays the geomagnetic sample points used for all SGSRs along with their corresponding semi-variogram parameters. The second column presents the generated SGSRs, and the third column shows the histogram distribution of each SGSR surface, including its mean and variance. The histograms confirm that the generated SGSRs follow a normal distribution, consistent with the original sample points.

**Fig. 11:**
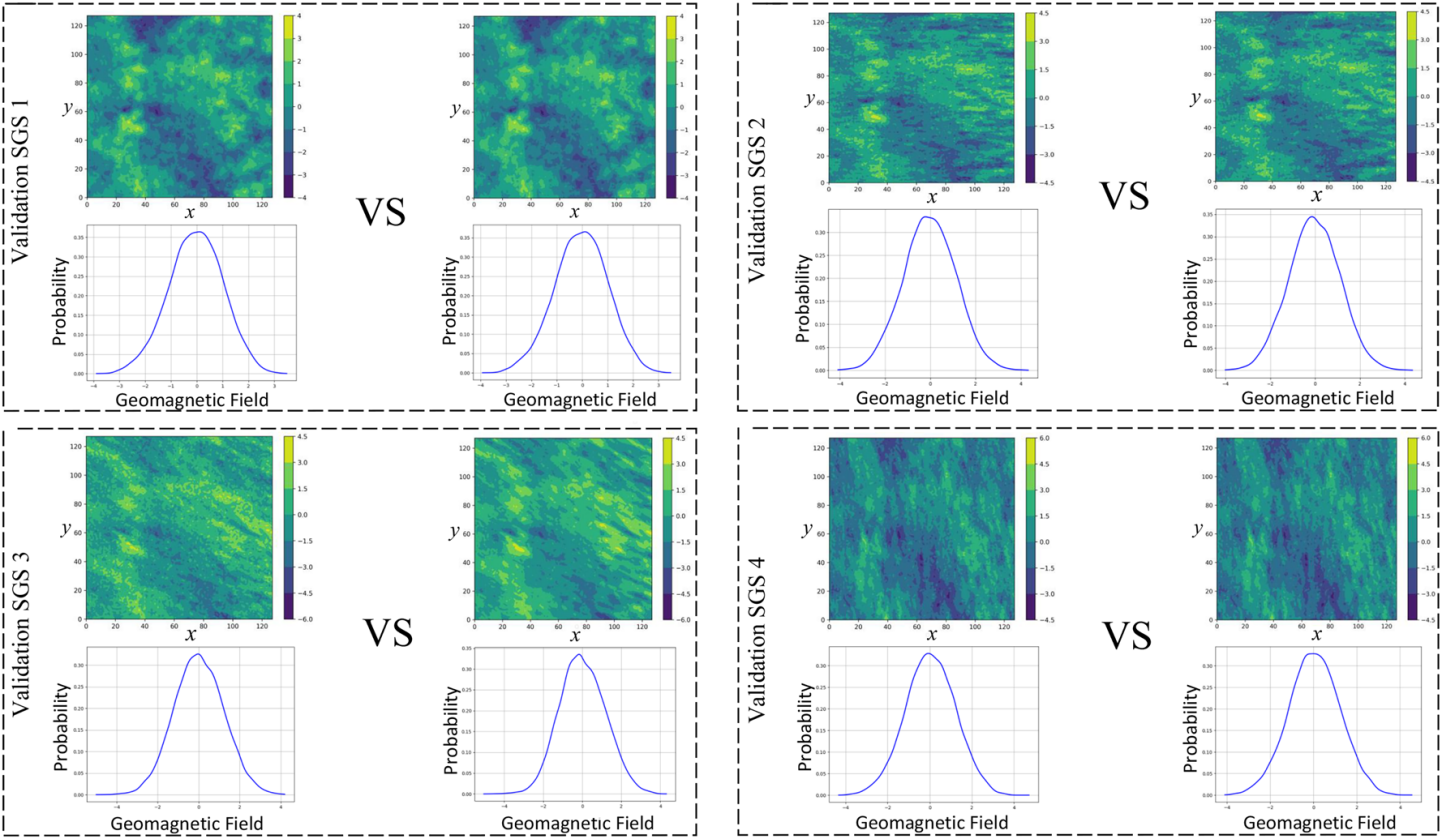
SGS estimate output by the encoder and its probability distribution diagram. For each test case, the first column shows the empirical distribution of true SGS surface values, and the second shows the distribution of SGS estimates produced by the decoder. Across the four cases, the distributions are not identical, yet their shapes and trends align closely, demonstrating the decoder’s effectiveness.

Next, we evaluated the encoder’s ability to estimate SGS values. The network parameters corresponding to the minimum training loss were saved, reloaded, and applied to inference on the 1360 test samples reserved for validation. The results are shown in Fig. 11. For each validation case, the first column displays the true SGS surface and its probability distribution, while the second column shows the encoder-generated SGS estimate and its associated geomagnetic value distribution.

As an illustrative example, four randomly selected validation samples were analyzed. The true SGS of the first sample exhibits a magnetic variation trend oriented from southwest to northeast, corresponding to an azimuth of $10^{\circ }$ east of north. Although subtle, the estimated SGS surface shows a consistent directional trend. For the second sample, both the true and estimated SGS surfaces align at $90^{\circ }$, with fully consistent orientations. The third and fourth samples have true directional angles of $120^{\circ }$ and $170^{\circ }$, respectively, while the corresponding SGS estimates exhibit northwest and southeast trends, matching the expected orientations.

These results—representative of the broader test set—show that the encoder captures the dominant spatial structure of the geomagnetic samples and reliably infers spatial continuity in SGS-estimated surfaces.

For the decoder component, the primary task is to infer semi-variogram parameters from the SGS estimates reconstructed by the encoder. The experimental results are summarized in Table 4, with four test datasets provided as illustrative examples. The differences between the labels and estimated parameters for Data 1–4 are less than $3^{\circ }$ in azimuth angle, the maximum range deviation is below 1 km, the anisotropy ratio differs by less than 0.1, and the nugget value error is under 0.01. These results indicate that the decoder successfully extracts the semi-variogram parameters from the encoder-generated SGS estimates.

**Table 4:** GMAS-CNN validation set parameter estimation.

Test data		Azimuth (Degree)	Range (km)	Anisotropy ratio	Nugget effect
Data1	Label	10	35	3.8	0
	Estimate	9.926	35.282	3.788	0.00268
Data2	Label	60	45	4.8	0.4
	Estimate	60.745	45.6375	4.7891	0.396
Data3	Label	90	15	2.6	0.2
	Estimate	90.4719	15.036	2.60494	0.20437
Data4	Label	170	15	2.0	0.3
	Estimate	168.483	15.0284	2.02	0.2998

#### Automated semi-variogram inference test for actual geomagnetic sampling points

**Fig. 12:**
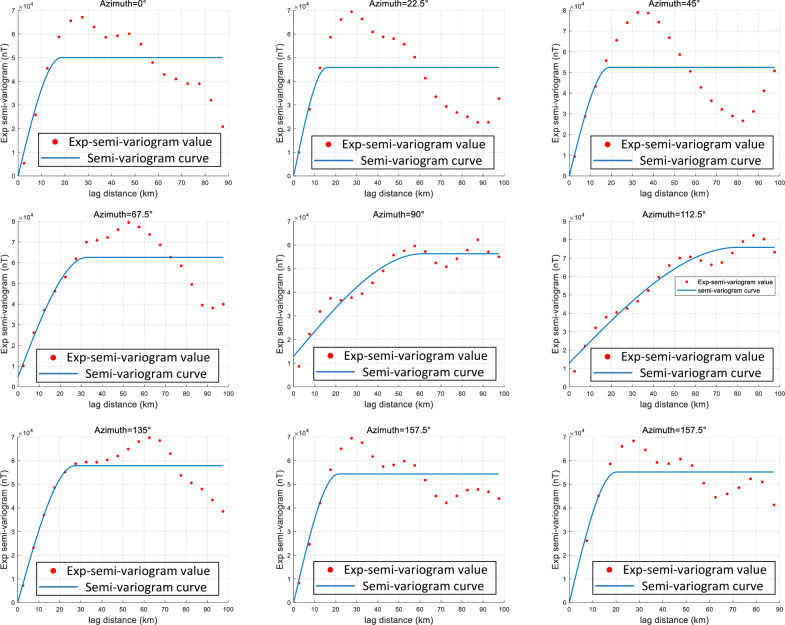
Calculation of the experience semi-variogram function from different angles. From the figure, the lag distance peaks at azimuth $90^{\circ }$ (approximately $80,\textrm{km}$) and begins to decline by $112.5^{\circ }$. This directional behavior constrains the admissible parameter range for the semi-variogram model.

To evaluate the accuracy of the proposed network, $100 \times 10$ initial geomagnetic sample points were used to infer their corresponding semi-variogram parameters. For validation, empirical semi-variograms were computed at angular intervals of $22.5^{\circ }$ from $0^{\circ }$ to $180^{\circ }$, as shown in Fig. 12. The results indicate that the maximum range occurs approximately within the interval [$90^{\circ }$, $112.5^{\circ }$]. At this orientation, the semi-variogram parameters are: anisotropy angle $112.5^{\circ }$, maximum range 83 km, anisotropy ratio 3.1, nugget value 0.2739, and sill value 1.1422. These results suggest that the geomagnetic sample points exhibit an anisotropy angle of approximately $112.5^{\circ }$.

**Fig. 13:**
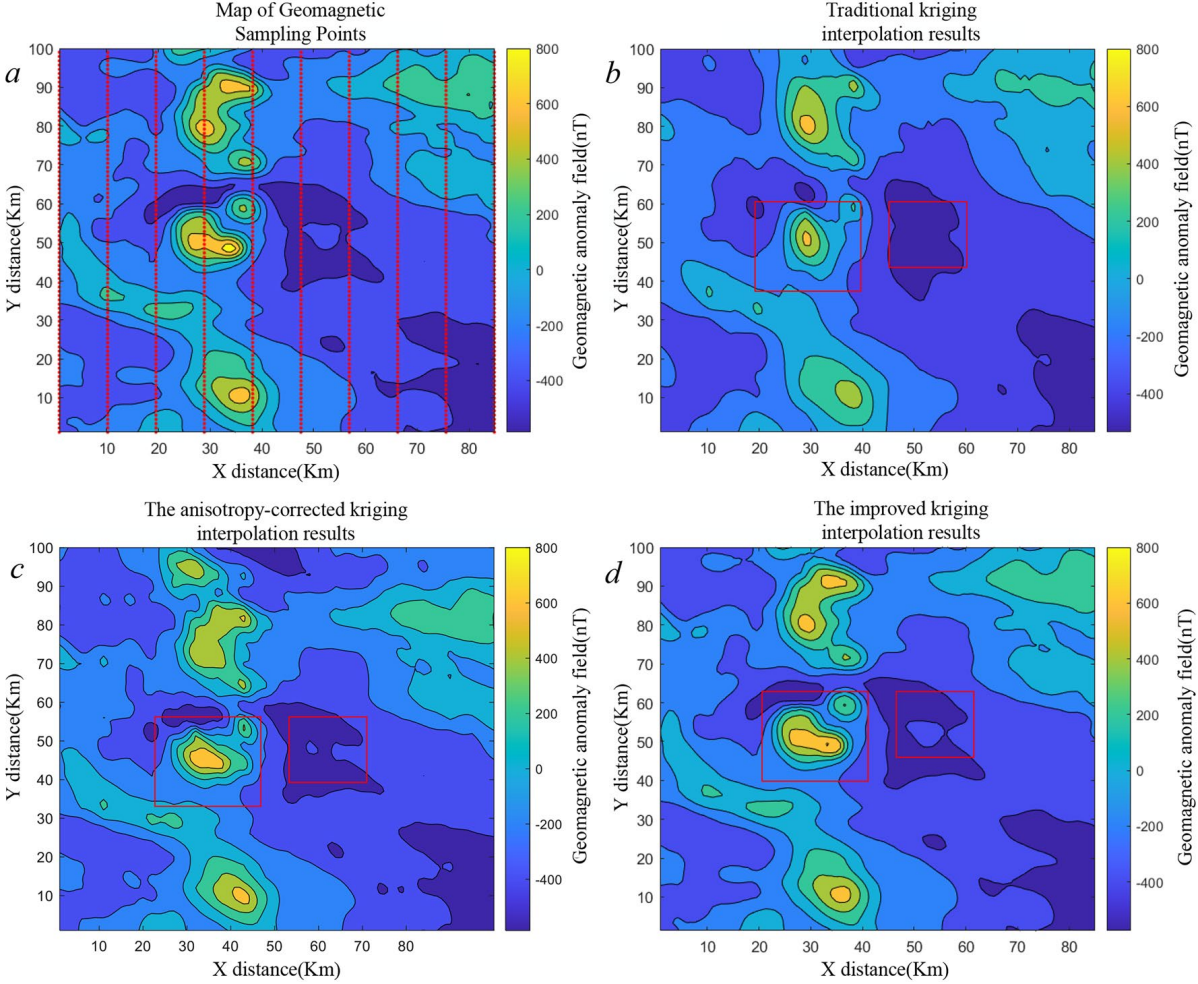
Interpolation results for geomagnetic sample points under ordinary kriging, anisotropy-corrected kriging and the improved kriging method proposed here. Panels: (**a**) sample locations; (**b**) ordinary kriging; (**c**) kriging with anisotropy-corrected; (**d**) the proposed improved method. In (**b,c**), hand-tuned semi-variogram parameters lack precision, introducing fine-scale errors; the contour lines within the red boxes are visibly distorted, whereas (**d**) mitigates these artifacts.

Geomagnetic sample point data were fed into the GMAS-CNN network, where the encoder enhanced semi-variogram information and the decoder inferred semi-variogram parameters. The inferred values for azimuth, maximum range, anisotropy ratio, nugget value, and sill are $103.4^\circ$, 78 km, 2.8, 0.153, and 0.89, respectively. As shown in Fig.13, the azimuth falls within [$90^\circ$, $112.5^\circ$], and the maximum range aligns well with expectations, further validating the effectiveness of the network.

#### Test of results of geomagnetic map construction

**Fig. 14:**
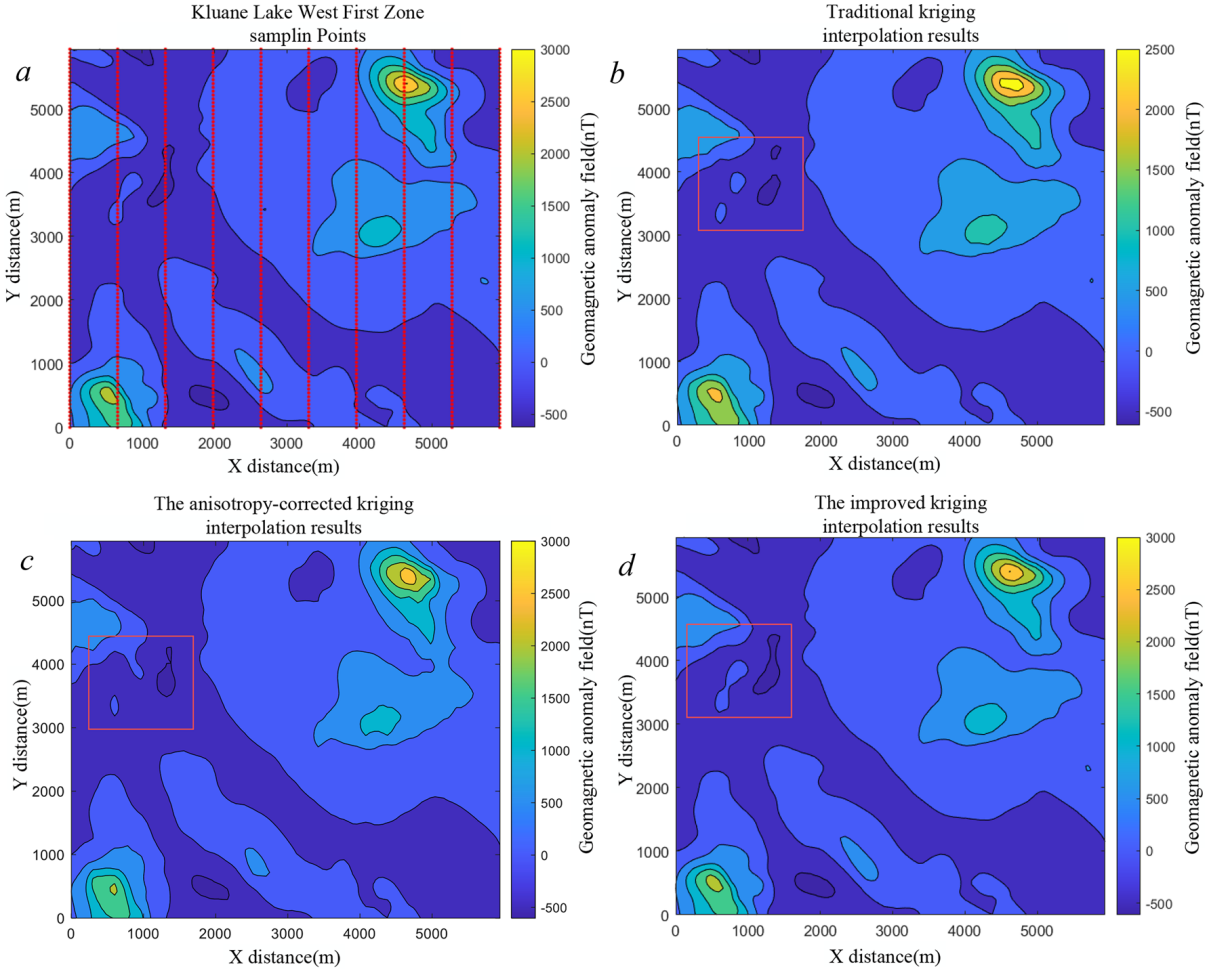
(**a**) Interpolation results for the Kluane Lake West First Zone sampling points. Panel (**b**) shows the geomagnetic map generated using ordinary kriging, panel (**c**) displays the map obtained with anisotropy-corrected kriging, and panel (**d**) presents the map constructed with the method proposed in this paper.

Ordinary and anisotropy-corrected kriging yield the maps in Fig. 14b,c after estimating the semi-variogram and performing kriging. In contrast, our pipeline first predicts semi-variogram parameters with GMAS-CNN, applies kriging to the geomagnetic samples, and then performs an inverse normal transformation to restore physical units; the resulting map appears in Fig. 14d. Ordinary kriging produces distorted contours, largely due to misestimated anisotropy angles, which induces substantial deviations from truth and degrades map accuracy. Kriging with anisotropy-corrected performs between ordinary kriging and our approach but remains sensitive to semi-variogram parameter accuracy. By comparison, the proposed method yields contours that more faithfully follow the true geomagnetic field, indicating higher interpolation fidelity.

Ordinary and anisotropy-corrected kriging yield the maps We further computed kriging- estimated variance maps for all three approaches (see Table 5). The proposed method and standard kriging yield largely consistent variance patterns. By contrast, the anisotropy-corrected variant reports smaller nominal variances, yet its interpolations are poorer because it relies heavily on manually specified semi-variogram parameters. This divergence between reported uncertainty and realized accuracy indicates that our method more reliably recovers the semi-variogram model parameters.

**Table 5:** Kriging estimated variance for ordinary kriging, anisotropy-corrected kriging, and the method proposed herein.

Method	Ordinary kriging	Anisotropy-corrected kriging	The improved kriging
Kriging variance estimation	0.014	0.01	0.013

Common metrics for evaluating geomagnetic interpolation include the mean error (ME), mean absolute error (MAE), and root mean square error (RMSE). Table 6 summarizes these metrics for geomagnetic maps generated using ordinary kriging, anisotropy-corrected kriging, and the proposed method. The results show that our approach achieves markedly higher interpolation accuracy, attributable to more precise estimation of semi-variogram parameters.

**Table 6:** Comparison of error between ordinary kriging interpolation, anisotropy-corrected kriging interpolation and kriging interpolation results proposed in this paper.

Method	ME (nT)	MAE (nT)	RMSE (nT)
Ordinary kriging	260.4138	10.3876	21.9779
Anisotropy-corrected kriging	194.1091	6.967	18.055
Improved kriging	193.0333	6.6259	17.7586

Based on the results in Table 6, the proposed algorithm achieves substantial improvements in geomagnetic map accuracy compared with ordinary kriging, with ME, MAE, and RMSE reduced by $25.76\%$, $36.22\%$, and $19.2\%$, respectively. The accuracy of the geomagnetic map constructed using the anisotropy-corrected method is comparable to that achieved by the method proposed in this paper. To further evaluate the general applicability of the method, two regions were selected from the publicly available Kluane Lake West Aeromagnetic Survey dataset in Canada (https://open.canada.ca/data/en/dataset/71fa28da-b1c9-cbf2-25c7-fd302c7643a3). We generated two datasets using the two sets of sample points and tested them on their respective test sets. The results from these two sets of test data are presented in Table 7. The table indicates that parameter biases for the Second Zone are relatively larger than for the First Zone. We conjecture that this discrepancy is associated with the geomagnetic distribution characteristics of the Second Zone.

**Table 7:** Parameter estimation for the Kluane Lake West Aeromagnetic Survey dataset in the GMAS-CNN network.

Test data		Azimuth (Degree)	Range (km)	Anisotropy ratio	Nugget effect
First Zone Data1	Label	30	25	1.6	0.2
	Estimate	30.336	24.917	1.573	0.2321
First Zone Data2	Label	110	45	2.8	0.2
	Estimate	109.569	45.6375	4.7891	0.396
Second Zone Data1	Label	30	25	1.6	0.2
	Estimate	35.107	28.814	1.905	0.40270
Second Zone Data2	Label	110	45	2.8	0.2
	Estimate	119.134	49.632	2.409	0.3785

The first and second zones were selected as two independent test sets to validate the model’s parameter estimation performance.

**Table 8:** Comparison of errors between ordinary kriging interpolation and the kriging interpolation results proposed in this paper for Kluane Lake West First and Second zones.

Interpolation zone	Method	ME (nT)	MAE (nT)	RMSE (nT)
First zone	Ordinary kriging	170.3338	16.8874	24.0973
	Anisotropy-corrected kriging	135.4077	13.0901	20.5671
	Improved kriging	133.2879	12.7631	19.3809
Second zone	Ordinary kriging	246.8333	15.6740	26.9266
	Anisotropy-corrected kriging	229.3199	18.7011	26.0908
	Improved kriging	220.0626	14.5297	24.0626

**Fig. 15:**
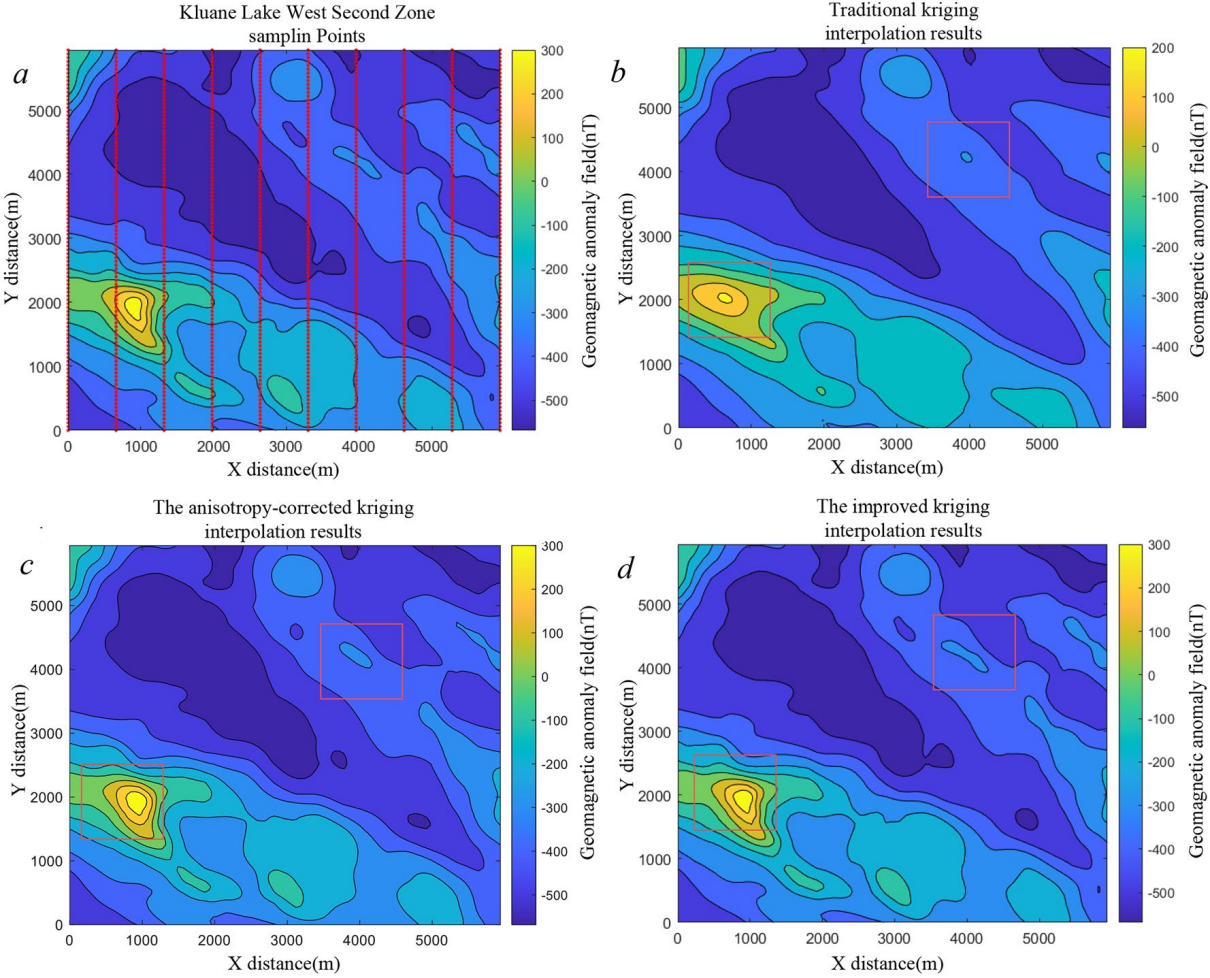
(**a**) Interpolation results for the Kluane Lake West Second Zone sampling points. Panel (**b**) shows the geomagnetic map generated using ordinary kriging, panel (**c**) displays the map obtained with anisotropy-corrected kriging, and panel d presents the map constructed with the method proposed in this paper.

The data were cropped and resampled, and interpolation was performed using both ordinary kriging, anisotropy-corrected kriging and the proposed approach. The resulting maps are shown in Figs. 14 and 15. In Fig. 14b, the ordinary kriging map exhibits discontinuous contour lines, while in Fig. 15b, local structural details are lost. Kriging methods with anisotropy-corrected provide superior trend fitting for the geomagnetic field; however, the accuracy of the semi-variogram obtained through manual experience is insufficient, resulting in geomagnetic maps with significant errors. By contrast, the maps generated using the proposed method preserve structural continuity and finer details.

The quantitative results are presented in Table 8. In Region 1, ME, MAE, and RMSE decreased by $21.75\%$, $12.58\%$, and $19.57\%$, respectively, whereas in Region 2, the reductions were $10.85\%$, $7.30\%$, and $10.64\%$. The comparatively smaller improvement in Region 2 is attributed to pronounced fluctuations in its geomagnetic values; conventional kriging tends to smooth these variations, thereby amplifying local interpolation errors. The anisotropy-corrected kriging method and the proposed approach exhibit comparable accuracy in geomagnetic map construction. However, the semi-variogram model parameters in our method are derived automatically, without reliance on manual tuning, making the process more efficient and fully automated.

All experiments were run on a workstation with two Intel Xeon Silver 4210R CPUs, 64,GB RAM, and four NVIDIA RTX3080Ti GPUs; training finished in approximately 31 minutes. Although slower than conventional kriging for geomagnetic map construction, the pipeline is fully automated and requires no human supervision.

#### Sensitivity to variogram parameters

The variogram dictates both smoothing and uncertainty in kriging interpolation. The nugget captures small-scale variability and elevates kriging variance; the sill defines the overall variance magnitude; the range controls the correlation length—larger values produce smoother maps—and anisotropy governs directional continuity. In our trans-Gaussian framework, these parameters are jointly inferred, which regularizes minor perturbations and mitigates their impact on predictions. Although a comprehensive perturbation analysis is left for future work, this qualitative examination highlights which parameters exert the greatest influence and clarifies their effects on model behavior.

## Conclusions

To enhance the accuracy of geomagnetic navigation, this paper addresses the precision of geomagnetic maps and proposes GMAS-K, a method for constructing high-resolution geomagnetic maps from sampling points. The key advantage of GMAS-K lies in its use of the GMAS-CNN network to directly infer the semi-variogram parameters of geomagnetic samples, thereby reducing errors introduced by manual parameter estimation. Experimental results demonstrate that the proposed method achieves at least a $10.64\%$ reduction in RMSE compared with ordinary kriging, as confirmed by evaluation metrics including ME, MAE, and RMSE. These improvements directly contribute to more accurate navigation and positioning.

While GMAS-CNN exhibits strong performance in constructing high-resolution geomagnetic maps, further improvements are possible. In this paper, the semi-variogram of geomagnetic samples was modeled using a spherical function. Extending the method to alternative semi-variogram models would require optimization of the network architecture. Moreover, constructing three-dimensional geomagnetic maps would demand richer datasets and deeper network structures to capture the added spatial complexity. Furthermore, a comprehensive perturbation analysis of the model’s sensitivity to semi-variogram parameters will be conducted in future work.

## Data Availability

The geomagnetic dataset analyzed in this study originates from the Kluane Lake West Aeromagnetic Survey. The data are publicly accessible via the Open Government Portal of Canada (https://open.canada.ca/data/en/dataset/71fa28da-b1c9-cbf2-25c7-fd302c7643a3).
